# The meaning of ‘life’ and other abstract words: Insights from neuropsychology

**DOI:** 10.1111/jnp.12065

**Published:** 2015-02-23

**Authors:** Paul Hoffman

**Affiliations:** ^1^ Neuroscience and Aphasia Research Unit (NARU)University of ManchesterUK; ^2^ Centre for Cognitive Ageing and Cognitive Epidemiology (CCACE)Department of PsychologyUniversity of EdinburghUK

**Keywords:** semantic cognition, concreteness, imageability, semantic aphasia, semantic control, semantic dementia

## Abstract

There are a number of long‐standing theories on how the cognitive processing of abstract words, like ‘life’, differs from that of concrete words, like ‘knife’. This review considers current perspectives on this debate, focusing particularly on insights obtained from patients with language disorders and integrating these with evidence from functional neuroimaging studies. The evidence supports three distinct and mutually compatible hypotheses. (1) Concrete and abstract words differ in their representational substrates, with concrete words depending particularly on sensory experiences and abstract words on linguistic, emotional, and magnitude‐based information. Differential dependence on visual versus verbal experience is supported by the evidence for graded specialization in the anterior temporal lobes for concrete versus abstract words. In addition, concrete words have richer representations, in line with better processing of these words in most aphasic patients and, in particular, patients with semantic dementia. (2) Abstract words place greater demands on executive regulation processes because they have variable meanings that change with context. This theory explains abstract word impairments in patients with semantic‐executive deficits and is supported by neuroimaging studies showing greater response to abstract words in inferior prefrontal cortex. (3) The relationships between concrete words are governed primarily by conceptual similarity, while those of abstract words depend on association to a greater degree. This theory, based primarily on interference and priming effects in aphasic patients, is the most recent to emerge and the least well understood. I present analyses indicating that patterns of lexical co‐occurrence may be important in understanding these effects.

## Background

Words are incredibly useful things. One of their functions is to act as labels for objects and actions that we perceive directly in our environment, allowing us to communicate those observations to others (e.g., ‘the cat sits on the mat’). But many words cannot be so closely tied to experiences in the external world and instead represent a complex array of less tangible concepts, including mental processes, values, social constructs, and relationships (e.g., ‘the collaboration was frustrating at times but ultimately worthwhile’). The distinction between concrete words that relate to tangible items and abstract words that do not is fundamental in linguistics and cognitive science. It is well established that abstract words are more difficult to process in a variety of situations. Healthy participants process abstract words less efficiently than their concrete counterparts, in word recognition (James, 1975; Strain, Patterson, & Seidenberg, 1995), recall (Jefferies, Frankish, & Lambon Ralph, 2006; Nelson & Schreiber, 1992; Romani, McAlpine, & Martin, 2008), comprehension (Holmes & Langford, 1976; Kounios & Holcomb, 1994; Schwanenflugel & Shoben, 1983), and production tasks (Goetz, Sadoski, Stricker, White, & Wang, 2007; Tyler, Voice, & Moss, 2000; Wiemer‐Hastings & Xu, 2005). It is also typically the case that neuropsychological deficits have a disproportionate effect on the processing of abstract words (e.g., Coltheart, 1980; Franklin, 1989; Goodglass, Hyde, & Blumstein, 1969; Hoffman, Rogers, & Lambon Ralph, 2011; Jefferies, Patterson, Jones, & Lambon Ralph, 2009; though some notable exceptions are discussed later).

In short, it is clear that there are important differences in the way in which cognitive and language systems deal with concrete and abstract words. In this review, I will consider current theories on the nature of these differences. I will focus particularly on the insights that have been gained through the study of patients with language impairments and on how these findings can be integrated with the rich set of functional neuroimaging data on how brain regions respond differently to each type of word. Theories of concreteness effects include hypotheses about the quantity and types of information that contribute to our knowledge of concrete and abstract words, about their differential reliance on linguistic context and the resulting demands they place on executive control processes, and about the ways in which the semantic relationships between words are organized. While each of these theoretical perspectives is valuable in understanding concreteness effects, collectively they also speak to a more general and fundamental issue in cognitive science: How conceptual knowledge is represented, organized, and regulated.

Before proceeding, I will briefly address an issue of terminology. The classification of a particular word as concrete or abstract is not always straightforward. Some words do not fit either definition particularly well and others shift when used in different ways (e.g., *literature*, which can refer to a physical collection of documents or, more abstractly, to a body of written work). There are two commonly used methods for adjudicating in such cases, both based on ratings. One approach asks participants to rate the concreteness of a word, which is defined as the degree to which it refers to a tangible entity in the world. The other is concerned with its imageability, defined as the ease with which the word elicits a mental image (Paivio, Yuille, & Madigan, 1968). Although in principle there is a distinction between these two constructs, in practice they turn out to be very highly correlated with one another (*r* = .83 in the norms of Paivio *et al*., 1968). As a consequence, most researchers treat concreteness and imageability as interchangeable and use either to distinguish between concrete and abstract words. This is the approach I have taken in this review: I use the term ‘concrete’ for words that are high in concreteness/imageability and ‘abstract’ for words low in concreteness/imageability (for an alternative view, see Kousta, Vigliocco, Vinson, Andrews, & Del Campo, 2011). It is also important to note that I use this binary terminology in the interests of linguistic parsimony. In reality, both concreteness and imageability are continuous variables and words can fall anywhere along this spectrum (e.g., *property* is less abstract than *circumstance* but less concrete than *beach*). Properties ascribed to ‘concrete’ or ‘abstract’ words are therefore true for words to varying degrees as a function of their concreteness.

## What are concepts made of?

Perhaps the most influential perspective on differences between concrete and abstract concepts is Paivio's *dual‐coding* theory (Paivio, 1986). Paivio noted that while both concrete and abstract concepts are used and experienced verbally, only concrete words are associated with sensory–perceptual information acquired through direct experience of their referents (for related perspectives, see Andrews, Vigliocco, & Vinson, 2009; Barsalou, Santos, Simmons, & Wilson, 2008). Paivio proposed that verbal and sensory–perceptual information were represented in separate semantic stores and that concrete words were benefited from *dual‐coding* theory in both stores, while abstract words were represented only in the verbal store. The non‐verbal store was assumed to hold representations of the sensory experiences associated with particular concepts, both through vision and through other sensory channels (e.g., what an object looks like and sounds like and how it moves). The verbal store was thought to comprise information about the linguistic and syntactic associations between words. More recent computational approaches have explored how semantic knowledge could be extracted from linguistic experience, by constructing semantic representations for words based on statistical regularities in their usage in language (Andrews *et al*., 2009; Griffiths, Steyvers, & Tenenbaum, 2007; Jones, Johns, & Recchia, 2012; Landauer & Dumais, 1997; Lund & Burgess, 1996). I discuss these approaches in more detail later.

Dual‐coding theory is an example of a more general belief that concrete words are associated with richer or more detailed semantic representations than abstract words. Jones (1985), for example, found that participants judged it easier to predicate (i.e., generate factual statements for) concrete concepts than abstract. Participants also produce more detailed definitions for concrete words (Goetz *et al*., 2007) and are able to generate more specific, item‐related semantic features for these words (Wiemer‐Hastings & Xu, 2005). This greater representational richness of concrete words is generally assumed to explain why concreteness effects are frequently observed in language processing tasks that place no explicit demands on semantic knowledge. For example, relative to abstract words, healthy individuals are faster to recognize concrete words presented visually (e.g., Balota, Cortese, Sergent‐Marshall, Spieler, & Yap, 2004; Evans, Lambon Ralph, & Woollams, 2012; James, 1975) and are faster to recognize and repeat them in the auditory modality (Tyler, Voice, & Moss, 2000). Such findings point to the role of word meaning in supporting word recognition and production processes, with the assumption that the richer representations of concrete words provide greater support (e.g., Welbourne, Woollams, Crisp, & Lambon Ralph, 2011).

Such effects are magnified in aphasic patients in whom orthographic or phonological processes are disrupted. The clearest example of this is the syndrome of phonological–deep dyslexia, an acquired reading disorder in which the mapping between orthography and phonology is severely impaired (Coltheart, 1980; Crisp & Lambon Ralph, 2006; Marshall & Newcombe, 1973). As a consequence, word reading in these patients depends critically on activation of semantic knowledge. A cardinal feature of phonological–deep dyslexia is more successful reading of concrete relative to abstract words, indicating that the richer semantic representations of concrete words provide more support to the reading process. Plaut and Shallice (1993) provided a detailed set of simulations of deep dyslexia within a connectionist computational modelling framework. They successfully simulated the concreteness effect by assuming that concrete words activated a greater number of units in the network's semantic system than did abstract words. Analogous findings have been observed in auditory processing in patients with deep dysphasia (Katz & Goodglass, 1990; Martin & Saffran, 1992).

The representational richness accounts propose that concrete words benefit from additional conceptual information that is not available to abstract words. This greater richness is an important source of support to the language system when orthographic or phonological processes are disrupted. What about damage to the semantic system itself? If representational richness was the *only* difference between concrete and abstract words, then one would expect that damage to the semantic system would always have a disproportionate effect on the weaker abstract words. While this is frequently the case (e.g., Franklin, 1989; Hoffman, Jefferies, & Lambon Ralph, 2010), there has also been a steady stream of neuropsychological cases who present with a reversal of this pattern: A semantic impairment that disproportionately affects knowledge of concrete objects (Breedin, Saffran, & Coslett, 1994; Cipolotti & Warrington, 1995; Macoir, 2009; Papagno, Capasso, & Miceli, 2009; Sirigu, Duhamel, & Poncet, 1991; Warrington, 1975; Warrington & Shallice, 1984). These cases are significant because they suggest that the cognitive and neural bases of concrete and abstract knowledge are at least partially separable, such that damage can disrupt concrete knowledge while leaving abstract words relatively spared. While these ‘reverse’ concreteness effects have been demonstrated across a range of tasks, they are often most striking when patients are asked to define words or to generate speech spontaneously. Warrington's (1975) patient AB, for example, produced appropriate definitions for 90% of abstract words but only 50% of concrete words (e.g., *supplication* – ‘making a serious request for help’ vs. *poster* – ‘no idea’). Macoir (2009) provided two examples of his patient SC's spontaneous speech that illustrate the apparent gulf between his knowledge of concrete and abstract concepts:

1. When there is a lot of snow on the roof of my house and my driveway, I remove the snow. I go on the roof of my house with my legs. I cannot tell you how I go up there. Also, I cannot tell you what I use to remove the snow. Also, for my driveway, I use another object that I cannot name. To defrost also, I do it but I do not remember the word for it.
2. I progressively discover that my personal thoughts develop my well‐being. I do not wish to prove that these thoughts are absolute. On the other hand, I wish to try them out daily to check if they can maintain this well‐being. What emerges gradually is the awareness of the symptom. What happens is that I am not able any more to increase my personal well‐being. When I wake up in the morning, my anxiety emerges immediately. The probability that I will make mistakes is huge. I constantly live in danger…

In the first sample, the patient fails to retrieve the names of several concrete objects while attempting to describe a commonplace event, yet in the second sample, he speaks fluently about his mental state, using a range of abstract terms. Loss of sensory–perceptual knowledge, in the visual modality particularly, has been proposed as the root cause in a number of reverse concreteness cases (Breedin *et al*., 1994; Macoir, 2009; Sirigu *et al*., 1991). In the above‐referenced cases, impairments to visual‐perceptual knowledge have been demonstrated directly within the domain of concrete objects. For example, Macoir's patient was much more accurate at verifying statements about the functional properties of objects (e.g., Is a knife used for cutting?) than about their perceptual qualities (Does a knife have a blade?). In addition, reverse concreteness effects sometimes occur in the context of severe deficits for particular categories of concrete item, such as animals, that are thought to draw heavily on visual experience and knowledge (Breedin *et al*., 1994; Papagno *et al*., 2009; Sirigu *et al*., 1991; Warrington & Shallice, 1984; though not in the case of Macoir, 2009). This interpretation of reverse concreteness effects therefore holds that they result from damage to an area of cortex involved in the representation of visual‐sensory aspects of semantic knowledge, which are particularly critical for concrete word knowledge.

Patients with reversed concreteness effects typically present with anterior temporal damage, often in the context of semantic dementia (SD). SD is a relatively rare neurodegenerative condition in which patients present with a progressive, profound, yet largely selective deterioration in semantic memory (Hodges, Patterson, Oxbury, & Funnell, 1992; Snowden, Goulding, & Neary, 1989). Many studies have tracked the deterioration of object concepts in this condition, which follows a reliable progression from the loss of fine discrimination between closely related items to an eventual failure to identify items even at a gross, superordinate level (Hodges, Graham, & Patterson, 1995; Rogers & Patterson, 2007; Woollams, Cooper‐Pye, Hodges, & Patterson, 2008). One account of these deficits holds that they are due to damage to representation of visual attributes coded in ventral temporal cortex and therefore are relatively selective for concrete concepts (Bonner *et al*., 2009). This view is consistent with findings that SD patients have particular difficulty in describing the perceptual properties of objects, relative to other characteristics (Lambon Ralph, Graham, Patterson, & Hodges, 1999), and that, as their disease progresses, they begin to show particularly poor knowledge for objects that depend heavily on their visual characteristics (Hoffman, Jones, & Lambon Ralph, 2012).

Another influential account proposes that atrophy to anterior temporal cortex in SD affects a multi‐modal conceptual knowledge store or ‘hub’ (Patterson, Nestor, & Rogers, 2007; Rogers *et al*., 2004). The hub is thought to use multiple channels of sensory‐motor input to form integrated concepts that link together the different forms of information associated with a particular concept (Hoffman, Evans, & Lambon Ralph, 2014; Lambon Ralph, Sage, Jones, & Mayberry, 2010). On this view, information pertaining to particular characteristics of a concept is distributed throughout the cortex (visual characteristics in occipitotemporal cortex, auditory characteristics in superior temporal regions, and so on) and the hub stores a representation of the concept as a whole that combines these various elements. While this conceptual binding function is critical for concrete concepts, which are associated with rich, multi‐modal sensory experiences, its role in representing the knowledge of abstract concepts is less clear (Meteyard, Cuadrado, Bahrami, & Vigliocco, 2012; Shallice & Cooper, 2013). One possibility is that because abstract words do not have rich multi‐modal associations, the hub does not play a key role in representing their meanings. As a consequence, damage to the hub would be expected to give rise to reverse concreteness effects. However, this intuitively appealing position is challenged by two lines of emerging evidence, one from larger‐scale studies of SD patients and the other from functional neuroimaging in healthy participants. I will deal with each in turn.

Until recently, reversed concreteness effects had only been reported in a small number of single‐case studies of SD and it was not clear whether this unusual pattern of performance was representative of the condition more generally. In recent years, a number of studies have investigated concreteness effects in larger groups of SD patients, with a complex pattern of results. Yi, Moore, and Grossman (2007) tested 12 SD patients on comprehension of verbs and found that as a group they displayed better comprehension of cognition verbs (abstract) than motion verbs (concrete) in a description‐to‐word matching task. In a follow‐up study, Bonner *et al*. (2009) found a similar effect in 11 SD patients, this time using a synonym judgement task (e.g., Is *life* most similar to *existence*, *position*, or *belief*?). These studies support the view that reverse concreteness effects are common in SD. In contrast, Jefferies *et al*. (2009) tested comprehension in their cohort of 11 SD patients using a different synonym judgement task but found no evidence of reverse concreteness effects. In fact, all 11 individuals displayed markedly *better* comprehension of concrete words than abstract. Why the discrepancy? The differing results could have been due to differences in the characteristics of the patients included in each study or in the properties of the stimuli used to test them.

We recently collated the stimuli from the Jefferies *et al*., Yi *et al*., and Bonner *et al*.'s studies, as well as other concreteness tests, and ran them all in a single set of seven individuals with SD (Hoffman & Lambon Ralph, 2011). The patients displayed a strong advantage for concrete words on the Jefferies *et al*.'s test while at the same time showing no difference between concrete and abstract words on the Yi *et al*. and Bonner *et al*.'s materials. This suggests that the discrepancies between studies are likely due to differences in stimuli. We found that the tests varied considerably in the degree to which their concrete and abstract words were separated along the imageability spectrum. The Jefferies *et al*.'s test employed a very strong manipulation of imageability – its abstract words were much less imageable than the abstract words used in other tests, and its concrete words were more imageable.^1^ Consequently, it delivered the clearest effects in the patients, and these strongly indicated poorer comprehension of more abstract words. Tests whose stimuli were less differentiated on the imageability scale produced weaker or absent effects.

**Figure 1:**
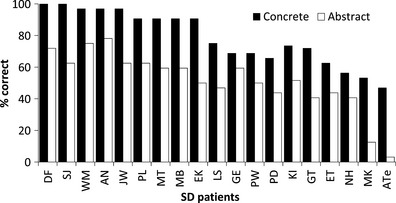
Synonym judgement performance in 19 semantic dementia (SD) patients. Results are presented for all 19 SD patients who participated in our research programme between 2003 and 2012. This comprises all of the patients reported by Jefferies *et al*. (2009), Hoffman and Lambon Ralph (2011), Hoffman, Jones, and Lambon Ralph (2013).

Figure 1 shows results on the Jefferies *et al*.'s synonym judgement test for the 19 SD patients who have participated in research in the Lambon Ralph laboratory in recent years (including the patients reported by Jefferies *et al*., 2009; Hoffman & Lambon Ralph, 2013). All of the patients showed an advantage for concrete words, ranging in size from 9% to 44%.^2^ The evidence from this large sample therefore indicates that the most common pattern in SD is for abstract word comprehension to be more severely impaired than concrete word comprehension. While it is certainly the case that reverse concreteness effects *can* occur in SD patients, these data suggest that they do not occur very often. In a follow‐up study with a subset of patients, we established that these results generalized to verbs as well as nouns and to associative as well as similarity‐based semantic relationships (Hoffman, Jones, & Lambon Ralph, 2013).

Reverse concreteness effects appear to represent an unusual deviation from the normal pattern in SD. What causes this deviation? Although it is impossible to be sure without direct comparison of patients presenting with each type of concreteness effect, Hoffman and Lambon Ralph (2011) proposed two possible causes. The first is that reverse concreteness patients may have had atypical premorbid experiences that influenced how concrete and abstract words were affected by the disease process. It is well known that the meanings of highly frequent or familiar words are less likely to become degraded in SD (Bozeat, Lambon Ralph, Patterson, Garrard, & Hodges, 2000; Funnell, 1995). For this reason, studies comparing concrete and abstract words typically employ stimuli that are carefully matched for lexical frequency. However, it is impossible to measure and control for the unique life experiences of each individual patient. It is possible that patients showing reverse concreteness effects had atypical backgrounds with a high level of exposure to abstract vocabulary, resulting in a predisposition to show less impairment for these words. We revisited the case reports of reverse concreteness patients and found that many such patients were highly educated professionals who would be expected to be highly literate and familiar with abstract terms (see Hoffman & Lambon Ralph, 2011). For example, Macoir's (2009) patient SC, who spoke so eloquently about his mental state, was a retired professor of psychology. Individual differences in lifetime exposure to and use of abstract words may therefore be a factor in understanding these unusual cases.

The second factor we considered was individual differences in the extent and distribution of cortical atrophy. Although SD is a homogenous disorder, with damage typically focused on the anterior temporal cortex, like any disease the precise pattern of damage varies across individuals. It is possible that reverse concreteness effects are a consequence of atypical presentations, with relative sparing of cortical regions critical for abstract word comprehension or disproportionate damage to areas involved in concrete word comprehension. To test this hypothesis, one would need to directly compare atrophy in patients showing typical and reversed concreteness effects. Unfortunately, no such studies exist at present. However, functional neuroimaging studies in healthy individuals have cast considerable light on which brain regions are preferentially involved in either concrete or abstract word processing. A number of studies have shown that the superior anterior temporal lobe (superior temporal sulcus and gyrus) is more strongly activated when participants process abstract words, relative to concrete (Binder, Desai, Graves, & Conant, 2009; Noppeney & Price, 2004; Sabsevitz, Medler, Seidenberg, & Binder, 2005; Wang, Conder, Blitzer, & Shinkareva, 2010). This area is closely linked with comprehension of speech and text, particularly at the sentence level (Humphries, Binder, Medler, & Liebenthal, 2006; Scott, Blank, Rosen, & Wise, 2000; Spitsyna, Warren, Scott, Turkheimer, & Wise, 2006). Its greater involvement in abstract words is therefore consistent with the dual‐coding view that comprehension of abstract words places strong demands on verbal aspects of semantic knowledge. If this area were unusually spared in a patient with SD, relative preservation of abstract word knowledge might result. Alternatively, we could consider areas of the ventral temporal cortex that are particularly involved in the processing and representation of the visual properties of objects (Chao, Haxby, & Martin, 1999; Martin, 2007). Areas of the ventromedial temporal cortex reliably show greater activation to concrete words in healthy individuals (Binder *et al*., 2009; Wang *et al*., 2010; Wise *et al*., 2000). The areas activated in these studies are typically posterior to the primary atrophic areas in SD, but atrophy can affect these areas, particularly in the later stages of the disease (Rohrer *et al*., 2009). An unusual preponderance of atrophy in this more posterior region might give rise to a particularly prominent concrete word deficit in some patients.

In recent years, there has also been growing interest in the anterior portions of the fusiform and inferior temporal gyri, an area I will refer to collectively as the ‘ventral ATL’. This region is severely atrophic and hypometabolic in SD patients (Galton *et al*., 2001; Nestor, Fryer, & Hodges, 2006), and dysfunction in this area is a strong predictor of the level of semantic impairment in patients (Mion *et al*., 2010). This association suggests that the ventral ATL is the site of the semantic ‘hub’ thought to represent supramodal conceptual information (Patterson *et al*., 2007; Rogers *et al*., 2004). Unfortunately, the majority of fMRI studies have provided no useful data on the ventral ATL, as the proximity of air‐filled sinuses makes it difficult to extract BOLD signal from this area reliably (Devlin *et al*., 2000; Ojemann *et al*., 1997; Visser, Jefferies, & Lambon Ralph, 2010). However, a number of recent studies have investigated this region using an optimized fMRI acquisition protocol designed to alleviate these technical difficulties (Embleton, Haroon, Morris, Lambon Ralph, & Parker, 2010). These studies have revealed strong activation of the ventral ATL when participants engage in semantic processing of words, pictures, and sounds (Binney, Embleton, Jefferies, Parker, & Lambon Ralph, 2010; Visser, Jefferies, Embleton, & Lambon Ralph, 2012; Visser & Lambon Ralph, 2011), in line with the findings in SD and with earlier positron emission tomography studies, which do not suffer from the same limitations as fMRI (e.g., Vandenberghe, Price, Wise, Josephs, & Frackowiak, 1996).

**Figure 2:**
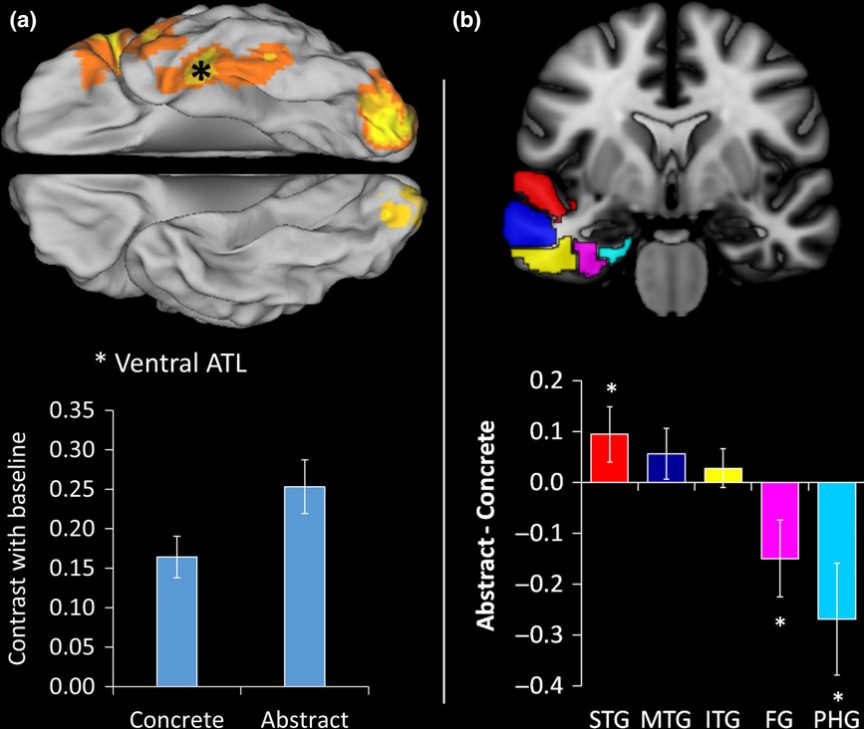
Activations to concrete and abstract words in the left anterior temporal lobe. Results are taken from an fMRI study conducted by Hoffman *et al*. (2015). Participants made synonym judgements to abstract and concrete words. The data presented in this figure are combined over two cueing conditions (see Figure 3 for details). (a) Activations to concrete and abstract word decisions in the ventral ATL (marked with an asterisk), relative to baseline. The cortical overlay indicates areas active for both word types. (b) Contrast of abstract minus concrete words in an anterior section of each temporal gyrus. STG, superior temporal gyrus; MTG, middle temporal gyrus; ITG, inferior temporal gyrus; FG, fusiform gyrus; PHG, parahippocampal gyrus.

In a recent fMRI study, we used the same acquisition technique to explore the responses of this region to concrete and abstract words (Hoffman, Binney, & Lambon Ralph, 2015). Participants completed a variant of the Jefferies *et al*.'s (2009) synonym judgement task while in the scanner. We found that the ventral ATL responded strongly to both types of word (see Figure 2a). This provides independent evidence for the idea that the ventral ATL, a critical area of damage in SD, is involved in representation of both concrete and abstract word knowledge. More generally, it suggests that the semantic hub region is not selective for concrete concepts. In fact, this region shows *greater* activity for abstract words, in line with the more severe deficits in abstract word comprehension in the majority of SD patients. The most parsimonious explanation for these results are that abstract words place greater demands on this shared neural substrate, as predicted by slower processing of abstract words in healthy individuals and by the idea that the representations of abstract words are less rich than those of concrete words.

We also found evidence of graded specialization for concrete and abstract words across the wider anterior temporal region. Figure 2b shows the contrast of abstract minus concrete words in an anterior section of each of the left temporal gyri. A gradual shift in responses is apparent. The superior and middle temporal gyri were more strongly activated by abstract words, while the fusiform and parahippocampal gyri displayed a preference for concrete words. Between these extremes, the inferior temporal gyrus showed similar activation for both word types. Therefore, while the ATL as a whole appears to be critical for both concrete and abstract words, there are more subtle gradations in response across the region. What are the implications of these findings? One possibility is that the ATL as a whole acts as a hub for semantic representation but that graded specialization emerges within this area as a function of differential connectivity with posterior sensory cortices (Binney, Parker, & Lambon Ralph, 2012; Hoffman *et al*., 2012; Plaut, 2002). On this view, the relative contributions of particular subregions of the ATL depend on the inputs they receive. The dorsolateral ATL is weighted towards auditory‐verbal aspects of experience, because this region receives strong connections from primary auditory processing apparatus of the posterior superior temporal sulcus (Binney *et al*., 2012; Fan *et al*., 2014; Pascual *et al*., 2015; Visser & Lambon Ralph, 2011). In contrast, the ventromedial ATL is strongly connected to posterior fusiform and parahippocampal areas of the ventral visual stream (Binney *et al*., 2012; Fan *et al*., 2014; Pascual *et al*., 2015), which mediate high‐level visual object processing, and thus displays relative specialization for visual‐perceptual aspects of knowledge (Visser *et al*., 2012). This graded specialization for verbal versus visual knowledge in the ATL, combined with the dual‐coding view that concrete and abstract words depend differentially on such knowledge, readily accounts for the pattern shown in Figure 2b.

The above discussion has focused on the relative importance of visual and verbal experience to concrete and abstract concepts, respectively, and on the established idea that abstract concepts lack the sensory information associated with their concrete counterparts. Researchers are now beginning to explore other aspects of experience that support abstract word comprehension. In an innovative study, Troche, Crutch, and Reilly (2014) asked participants to rate the relevance of various aspects of experience, such as time, space, thought, and emotion, to particular words (see also Crutch, Williams, Ridgway, & Borgenicht, 2012). They found that while concrete words were invariably strongly associated with perceptual sensation (as investigated in detail by Gainotti, Ciaraffa, Silveri, & Marra, 2009; Hoffman & Lambon Ralph, 2013), other aspects of experience were more salient for abstract words. In particular, they found that some abstract words were strongly linked to experience of emotions, morality, and social interaction, while others were associated with time, space, and quantity. Other authors have argued in parallel that emotional content plays an important role in representation of abstract concepts (Kousta *et al*., 2011). Interestingly, recent fMRI studies have shown that areas of the rostral anterior cingulate implicated in emotion processing are also engaged by processing of abstract words, as a function of their emotional content (Skipper & Olson, 2014; Vigliocco *et al*., 2014).

Less is known at present about the neural basis of the proposed contribution of quantity‐based information to abstract concepts, although one recent exploratory study probed neural responses to the abstract words *arithmetic* and *convince* (Wilson‐Mendenhall, Simmons, Martin, & Barsalou, 2013). The authors found that an area of intraparietal sulcus involved in representation of numerical magnitudes was activated when participants processed *arithmetic*, while social processing regions were more activated by *convince*. These recent studies suggest that knowledge for abstract words may depend on a distributed set of brain areas associated with aspects of knowledge beyond the sensory and linguistic. They also raise the intriguing possibility that particular forms of brain damage could lead to selective deficits for particular classes of abstract word, analogous to those observed for particular categories of concrete object (Capitani, Laiacona, Mahon, & Caramazza, 2003). Selective deficits for socially relevant words, relative to animal concepts, have been reported in patients with frontotemporal dementia, although these have not been directly contrasted with other types of abstract word (Zahn *et al*., 2009).

To summarize, research on the representational basis of concrete and abstract words has reached two main conclusions. The first is that we tend to have richer and more detailed knowledge of concrete words than we do of abstract words. This difference in representational richness can account for the large concreteness effects often observed in aphasic patients and for the typical pattern of knowledge degradation in SD, in which comprehension of abstract words is more severely impaired. The second conclusion is that concrete and abstract words differ in the types of information that contribute to their representation. Traditionally, researchers have focused on differential reliance on verbal versus sensory experience, although recent work has begun to highlight the roles that emotional and quantity‐based information plays in supporting some abstract words. The neural basis of such specialization is also beginning to be uncovered. This is most clear in the temporal lobe, where dorsolateral regions show greater activity when participants comprehend abstract words while ventromedial areas respond preferentially to concrete concepts. Interposed between these extremes, the central ‘hub’ region of the ventral ATL responds strongly to both word types, mirroring the tight coupling of concrete and abstract knowledge deficits in SD.

## The role of context and executive regulation

In the previous section, I considered the types of information that contribute to our understanding of concrete and abstract concepts. Next, I will consider the key role that context plays in shaping the meanings of abstract words and the consequences of this contextual variability for patients with deficits in the executive regulation of knowledge. We often think of words as invariant tokens of meaning, but in reality, their meanings frequently shift in different situations. Take the word *life* as an example. This can be used to refer to a general property of biological organisms (‘The insect clung to life’), as an umbrella term for the organisms themselves (‘Life on earth is fragile’), as a term for the span of a person's existence and its development over time (‘She entered a new phase in her life’), as a more abstract property denoting animacy or vigour (‘The play came to life in the second act’), or as an indeterminate sentence given to an offender (‘The judge gave him life’). These different uses are sometimes termed ‘senses’, and there are subtle but important differences between each of them.^3^ The correct interpretation in any particular situation is determined by the context in which the word is being used. For effective comprehension of language, the semantic system therefore has to shape its interpretation of the meaning of each word according to the particular context in which it occurs. This process is particularly demanding for words whose meanings are highly variable across contexts, especially when the context is weak, ambiguous, or absent altogether (as is typically the case in psychological experiments).

What determines the degree of contextual variability associated with each word? One might expect the meanings of concrete words to be less contextually varying because their meanings are tied to a fixed class of objects or events in the environment. Conversely, the tendency for abstract words to refer to less well‐defined, intangible experiences or properties may allow for greater variation across contexts. Schwanenflugel and colleagues first investigated these ideas in a series of seminal studies in the 1980s (Schwanenflugel, 1992; Schwanenflugel, Harnishfeger, & Stowe, 1988; Schwanenflugel & Shoben, 1983). They found that processing advantages for concrete words in lexical decision and sentence reading could be eliminated by placing the stimuli in meaningful contexts. They attributed these effects to differences in the *context availability* of concrete versus abstract words, which they measured by asking participants to rate the ease with which they could imagine a context in which the word might appear. Context availability was lower for abstract words, and this factor proved to be a strong predictor of performance. Schwanenflugel *et al*. concluded that abstract words were more difficult to process because participants found it hard to place them in a meaningful context and that these effects were ameliorated when the experimenter provided such a context explicitly. Although work on context availability proved fruitful and influential, its reliance on subjective ratings made it difficult to understand the root cause of the effect. Are contexts less available for abstract words because they simply occur in fewer contexts than concrete words? Or are there a great many competing contexts in which an abstract word could occur, such that no single context comes to mind strongly?

In the years since Schwanenflugel *et al*.'s work, a number of sophisticated computational techniques have been developed which permit the contextual usage of words to be analysed formally in large corpora of real language data (Andrews *et al*., 2009; Griffiths *et al*., 2007; Jones *et al*., 2012; Lund & Burgess, 1996). Latent semantic analysis is one such technique, which uses lexical co‐occurrence statistics to represent the relationships between words in terms of similarity in the contexts in which they are used and, in turn, to estimate the relatedness of the contexts themselves based on overlap in the words they contain (Landauer & Dumais, 1997). We recently used this technique to formally investigate the degree of contextual variability associated with particular words (Hoffman, Lambon Ralph, & Rogers, 2013). We took all of the contexts in which a given word was used and measured the average similarity of those contexts with one another, and we termed this quantity as the word's *semantic diversity*. We found substantial variation in this quantity across words. Some words appeared in a restricted, inter‐related set of contexts and consequently had low semantic diversity values (e.g., *spinach*, which typically only occurs in contexts related to cooking and eating, had a value of 0.99). Other words appeared in a wider range of disparate contexts and consequently had high values (e.g., *life* had a value of 2.13, with the maximum possible values being around 2.4 for function words like *also*, *which*, and *from*, which can be used in any context). Importantly, we found a strong negative correlation between semantic diversity and imageability, indicating that abstract words tend to be used in a wider variety of different contexts than do concrete words. This finding might seem at odds with the established view that concrete words have richer semantic representations. However, the higher semantic diversities of abstract words indicate only that they have greater variability in *linguistic* usage. It does not imply that they are associated with the multi‐modal non‐verbal experiences that are thought to enrich the representations of concrete terms. In addition, because there may be many different contexts in which an abstract word could potentially be used, its strength of association with any one context may be very weak. This view is consistent with Schwanenflugel and Shoben (1983) and Schwanenflugel *et al*. (1988) findings that participants find it harder to think of a specific context in which they could use a word when they are presented with abstract words.

What are the cognitive consequences of the greater semantic diversity of abstract words? As detailed above, the wide array of contexts associated with abstract words is likely to bring with it variations in the meanings of those words and the semantic knowledge associated with them. This means that, in any particular situation, only a subset of the information associated with the word is currently relevant and other aspects are not (e.g., in the phrase ‘signs of life’, the biological and medical aspects of *life* are highly relevant but those relating to a person's development over their lifespan are not). Effective comprehension is therefore thought to require input from executive processes that provide top‐down regulation of knowledge, often referred to as *semantic control* (Jefferies & Lambon Ralph, 2006). It is well known that executive processes make an important contribution to semantic processing, by selecting among competing meanings or aspects of knowledge and by biasing knowledge retrieval towards the requirements of current situation or task (Badre & Wagner, 2002; Gold *et al*., 2006; Rodd, Davis, & Johnsrude, 2005; Thompson‐Schill, 2003). Much attention has been focused on the role of left inferior prefrontal cortex in these top‐down executive influences, although recent studies indicate that posterior middle temporal gyrus and inferior parietal cortex also make important contributions (Jefferies, 2013; Noonan, Jefferies, Visser, & Lambon Ralph, 2013; Whitney, Kirk, o'Sullivan, Lambon Ralph, & Jefferies, 2012). The more variable meanings of abstract words suggests that semantic control may be particularly important for the comprehension of these words. Indeed, regions of inferior prefrontal cortex associated with semantic control are frequently more active when healthy participants process abstract words in the scanner, relative to concrete words (Binder *et al*., 2009; Noppeney & Price, 2004; Wang *et al*., 2010).

Over the past few years, we have investigated concreteness effects in a group of stroke patients who have established deficits in semantic control. These patients were originally identified by Jefferies and Lambon Ralph (2006) on the basis that they (1) were chronically aphasic following stroke and (2) had a central semantic impairment (evidenced by impairment on verbal and non‐verbal semantic association tasks). This profile was termed *semantic aphasia* (a term originally coined by Head, 1926). The patients had damage to areas of prefrontal, posterior temporal, and inferior parietal cortex now known to be involved in semantic control, but not to the anterior temporal regions which are implicated in SD and involved in representation of conceptual information (Noonan, Jefferies, Corbett, & Lambon Ralph, 2010). Accordingly, their semantic deficits were different in character to those observed in SD and clearly indicated problems with the executive regulation of semantic knowledge. Unlike SD patients, patients with semantic aphasia (SA) are strongly influenced by task demands, performing particularly poorly on tasks that require them to detect weak associations between concepts (e.g., they can match *bank* with *money* but not with *river*) or to inhibit strong but task‐irrelevant associations (Jefferies & Lambon Ralph, 2006; Noonan *et al*., 2010). Often their verbal responses indicate that they have retrieved information that is semantically linked with the stimulus but failed to select the required information for the task at hand (e.g., saying ‘nuts’ when asked to name a picture of a squirrel). They are often able to give appropriate responses when these are tightly constrained by the task but have difficulty in more open‐ended situations (Corbett, Jefferies, & Lambon Ralph, 2011; Jefferies, Patterson, & Lambon Ralph, 2008). Finally, they exhibit refractory effects in their semantic processing, which are thought to indicate a problem with access to semantic knowledge, rather than storage (Jefferies, Baker, Doran, & Lambon Ralph, 2007; Warrington & Cipolotti, 1996; Warrington & Shallice, 1979). In short, the behaviour of patients with SA is consistent with the idea that their semantic knowledge, coded in anterior temporal cortex, is intact but that they lack the semantic control processes necessary to regulate their use of this knowledge in line with current situational demands.

**Figure 3:**
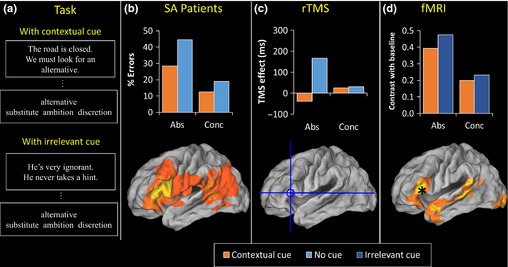
Convergent evidence linking left inferior prefrontal cortex with executive demands of comprehending abstract words. (a) Examples of task used in each study. Decisions were made following the presentation of a cue that placed the judgement in a context (top), a cue that was irrelevant to the judgement (bottom) or with no cue (not shown). (b) Performance of six semantic aphasic patients reported by Hoffman *et al*. (2010). Contextually cued trials are contrasted with no cue trials. The cortical map shows a lesion analysis for the group, with yellow indicating areas damaged in the greatest number of patients. (c) Effects of repetitive transcranial magnetic stimulation (TMS) to left inferior prefrontal cortex, as reported by Hoffman *et al*. (2010). Contextually cued trials are contrasted with no cue trials. The crosshairs indicate the target region for the TMS. (d) fMRI activations in left inferior prefrontal cortex, as reported by Hoffman *et al*. (2015). Results are taken from a region of interest centred on the target from the TMS study and indicated by an asterisk. The cortical overlay indicates regions activated by the task as a whole.

Hoffman *et al*. (2010) tested six SA patients on comprehension of concrete and abstract words, using the synonym judgement task described earlier. We found poorer comprehension of abstract words in every case. Critically, we found that performance was boosted when we provided the patients with additional contextual information that constrained their semantic processing of the word in question. Specifically, we preceded each semantic judgement with a sentence that placed the judgement word in a specific, meaningful context (see Figure 3a for examples). The manipulation improved performance, benefiting abstract words significantly more than concrete words (see Figure 3b). A third condition, in which decisions were preceded by an irrelevant cue, had a detrimental effect on performance. We suggested that the patients found it particularly difficult to make decisions about abstract words because, in the absence of context, these words activate a wide range of semantic information and the patients lacked the semantic control resources necessary to focus on the appropriate information. The contextual sentences were beneficial because they provided additional bottom‐up support that directed the semantic system towards a particular, contextually appropriate subset of information.

More direct evidence for this hypothesis was obtained in a later study in which we investigated which psycholinguistic properties influenced 13 SA patients' performance on the synonym judgement task (Hoffman *et al*., 2011). We entered three variables – word frequency, imageability, and semantic diversity – into a multiple regression model that predicted performance on individual trials of the test. Semantic diversity, the measure of the degree to which a word is used in a diverse set of contexts, was the single best predictor. Patients performed more poorly on the more semantically diverse words, supporting the idea that words with highly diverse meanings caused greater problems for their damaged semantic control mechanisms. Conversely, semantic diversity was *not* a significant predictor of performance in patients with SD, indicating that while both sets of patients have difficulty in understanding abstract words, the underlying causes of this deficit are different. Interestingly, semantic diversity is also a significant predictor of reaction times when healthy older adults complete the synonym judgement test (Hoffman, Lambon Ralph, & Rogers, 2013). This factor may therefore be important for understanding why healthy individuals, as well as patients with SA, are less efficient at comprehending abstract words.

More recently, other techniques have been used to obtain corroboratory evidence for the role of prefrontal executive control processes in abstract word comprehension. Hoffman *et al*. (2010) used repetitive transcranial magnetic stimulation (TMS) to disrupt activity in the left inferior frontal gyrus (IFG; specifically, BA45) of healthy individuals. Following TMS, participants were around 150 ms slower to comprehend abstract words in the synonym judgement task (see Figure 3c). No such effect was observed for concrete words. Importantly, the effect for abstract words was entirely eliminated when these were preceded by a meaningful sentence context, supporting the view that the critical role of prefrontal cortex in comprehending abstract words is related to the additional executive demands of regulating their variable meanings.

As described in the previous section, we have also used distortion‐corrected fMRI to investigate concrete and abstract word comprehension (Hoffman *et al*., 2015). In this study, each semantic judgement was preceded by a cue that participants read silently. On half of the trials, the cue placed the judgement word in a meaningful context, while on the other half, the cue was irrelevant to the judgement (see Figure 3a for examples). As shown in Figure 3d, the left IFG exhibited stronger activation for abstract words, as seen in many previous neuroimaging studies (e.g., Binder *et al*., 2009; Noppeney & Price, 2004; Wang *et al*., 2010). However, we also found that activation was reduced in this area when judgements were made with contextual support, suggesting that contextual constraints reduced the need for the executive control functions supported by this area. This result was in stark contrast to ATL regions: These areas showed *increases* in activation when contextual information was provided. Taken together, these results suggest a division of labour between IFG and ATL, whereby the ATL is maximally involved in contextually enriched language processing while the executive processes of the IFG are most critical when the semantic content of the stimulus is weak or ambiguous. For the present discussion, the most interesting outcome of this research is the observation that different cortical regions can show similar concreteness effects while responding to other semantic manipulations (i.e., context) in opposite ways. This indicates that the cognitive and linguistic factors underlying concreteness effects are multi‐faceted. These findings parallel those of neuropsychological studies, in which patients with SD and SA both demonstrate particular difficulties in comprehending abstract words, but only in the case of SA can this deficit be attributed to the higher semantic diversity of those words (Hoffman *et al*., 2011).

In summary, psycholinguistic studies indicate that abstract words are more likely to be used in a wide variety of linguistic contexts and consequently have more variable meanings. This greater semantic diversity results in difficulty in understanding abstract words for SA patients who have deficits in the executive regulation of their semantic knowledge (i.e., semantic control), particularly when context is not available to guide their interpretation. Convergent neuroimaging and stimulation studies suggest that it is this demand for semantic control that results in greater activation in left IFG for abstract words. Looking beyond IFG, it has become clear that the posterior middle temporal gyrus and intraparietal sulcus are also involved in the executive regulation of semantics (Jefferies, 2013). There is little evidence for differential involvement of these areas in the processing of concrete versus abstract words, however, and it is not clear why this is the case. Neuropsychological studies have the potential to provide further insights in this respect. Although the majority of patients with SA (including all those studied by Hoffman *et al*., 2010) have prefrontal damage, occasional cases present with a similar behavioural profile in the context of selective temporoparietal damage (Noonan *et al*., 2010). Better understanding of concreteness effects in these individuals could shed light on the contribution of posterior elements of the semantic control network to abstract word processing. In addition, temporoparietal atrophy is associated with language deficits in the syndrome of logopenic progressive aphasia (Gorno‐Tempini *et al*., 2008) but little is known at present about the fate of concrete versus abstract word processing in these patients.

## Differential frameworks in the structure of concrete and abstract concepts

Finally, I consider a new perspective on concrete–abstract differences that has emerged over the past 10 years based on innovative neuropsychological experiments. The *differential representational frameworks* hypothesis holds that concrete and abstract words differ in the way in which the relationships among concepts are organized (Crutch & Warrington, 2005). Concrete words are thought to be organized primarily in terms of semantic similarity (e.g., *dog* with *wolf*) while associative relationships (e.g., *theft* with *punishment*) are thought to be a more salient factor in the organization of abstract concepts. Evidence for this view has come primarily from studies of patients with semantic refractory access deficits (Crutch, Ridha, & Warrington, 2006; Crutch & Warrington, 2005, 2010). These patients are severely aphasic, typically following stroke, and experience interference between semantically related concepts (Warrington & Cipolotti, 1996; Warrington & Shallice, 1979). This interference has been probed most commonly using a spoken‐to‐written word matching task. Patients were presented with an array of four written words. The experimenter said one of the words aloud, and the patient was asked to point to that word in the array. For healthy subjects, this is a trivially easy task that can be accomplished purely through phonological‐to‐orthographic conversion. In these patients, however, the conversion process is impaired to such an extent that the patients are highly reliant on the semantic activation elicited by the spoken word to guide their choice. As a consequence, patients are liable to confuse words with closely related semantic representations and this leads to a characteristic pattern of performance whereby patients make more errors for arrays composed of semantically related words (e.g., *horse*, *cow*, *goat*, *sheep*) than for unrelated words (e.g., *horse*, *train*, *chair*, *tree*), as well as showing deteriorating performance when the same words are probed rapidly and repeatedly. These unusual patients therefore provide a unique opportunity to investigate how semantic relationships among different types of word are organized.

Crutch and Warrington (2005) reported a detailed investigation in one such patient, AZ. AZ was presented with arrays of concrete words that either belonged to the same category or were selected from different categories but shared semantic associations (see Figure 4a for examples). She showed a large interference effect for the same‐category arrays (i.e., performance was impaired, relative to a control condition in which the words in the array were unrelated) but no interference for the associated arrays. A different pattern emerged when AZ was tested on abstract words. For these, she showed interference effects for arrays composed of associated words but no effect for arrays of synonymous words. Crutch and Warrington suggested that the semantic representations of concrete words were organized by similarity, such that similar items from the same semantic category interfered with one another. In contrast, similarity in meaning appeared to be less influential in the organization of abstract words, with verbal associations critically important for these. These effects were later replicated in other patients (Crutch *et al*., 2006; but see Hamilton & Coslett, 2008), and corroboratory evidence has been sought using other methods, including analysis of errors and priming effects in deep dyslexic patients (Crutch, 2006; Crutch & Warrington, 2007). Recent studies have also used an odd‐one‐out detection paradigm with healthy participants, in which participants are asked to detect a semantically anomalous word within an array of related words (Crutch, Connell, & Warrington, 2009; Crutch & Jackson, 2011). For concrete words, participants were faster to spot the odd‐one‐out when the other words were semantically similar, rather than associated. For abstract words, the reverse was true.

**Figure 4:**
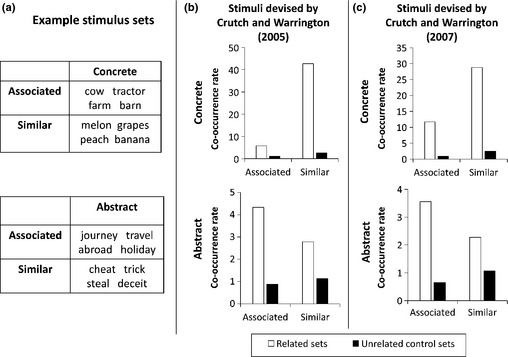
Lexical co‐occurrence rates for concrete and abstract words with different semantic relationships. (a) Examples of stimulus sets used by Crutch and colleagues to investigate similarity‐based and associative semantic relationships. (b, c) Lexical co‐occurrence rates in the British National Corpus for the stimulus sets used by Crutch and Warrington (2005, 2007). Co‐occurrence rates were calculated by computing how often each pair of words in each stimulus set co‐occurred in the corpus (within a 100‐word window) and dividing this by the expected co‐occurrence rate if co‐occurrences occurred by chance alone. This controls for the fact that higher frequency words are more likely to co‐occur by chance, even if their distributions are unrelated (Juteson & Katz, 1991).

The differential frameworks hypothesis holds that concrete and abstract words differ in how their meanings are structured and linked with one another. How compatible is this with the more established view that concrete and abstract words depend differentially on sensory and verbal knowledge? This depends to some extent on exactly how similarity‐based and associative relationships are defined. Crutch and Warrington (2010) defined similarity for concrete words as ‘tangible, directly perceived items that can be grouped under a common taxonomic category’. This is consistent with the popular view that knowledge for a concrete item can be usefully captured in terms as a list of its basic properties or semantic features (Cree & McRae, 2003; Devlin, Gonnerman, Andersen, & Seidenberg, 1998; Garrard, Lambon Ralph, Hodges, & Patterson, 2001; Tyler, Moss, Durrant‐Peatfield, & Levy, 2000; Vinson, Vigliocco, Cappa, & Siri, 2003; Zannino, Perri, Pasqualetti, Caltagirone, & Carlesimo, 2006). There is strong evidence from such feature listing studies that items from the same taxonomic category share many semantic features and this seems to be particularly true of perceptual features (Dilkina & Lambon Ralph, 2012). In other words, the strong semantic relationships between ‘similar’ concrete words may arise from the fact that their referents share many of the same perceptual features. This factor is unlikely to be important for abstract words, as these are not strongly associated with perceptual features. Indeed, when participants are asked to generate semantic features for abstract words, these are generally impoverished and the features often refer to contexts in which the concept could occur, rather than intrinsic properties (Wiemer‐Hastings & Xu, 2005).

Conversely, the definition of association is not related to features; instead, Crutch and Warrington define associatively related words as ‘items that can be drawn together to form a unitary, cohesive, real‐world, or imaginable context to which each item contributes some content’. This type of relationship is more compatible with an alternative perspective on semantic knowledge, which holds that the meanings of words are related to the degree that they co‐occur in similar linguistic contexts (Firth, 1957; Griffiths *et al*., 2007; Landauer & Dumais, 1997; Lund & Burgess, 1996; Rohde, Gonnerman, & Plaut, 2006). This approach emphasizes the role of knowledge derived from verbal experience, in the form of lexical co‐occurrences, in determining associative semantic relationships. One might expect abstract words, with their greater dependence on verbal experience, to be particularly influenced by this form of semantic organization.

To investigate the role of lexical co‐occurrence in these different forms of semantic relationship, I analysed how often words from the stimulus sets used by Crutch and Warrington (2005, 2007) co‐occurred in a large language corpus (the British National Corpus; British National Corpus Consortium, 2007). I divided the corpus into windows of 100 words and computed how often words held to share similar or associative semantic relationships co‐occurred in the same window. The results are shown in Figure 4, with data from two separate sets of materials presented side‐by‐side to illustrate the highly consistent findings. The black bars show co‐occurrence rates for the control stimuli – that is sets of words not thought to share any semantic relationship. These were all close to one, indicating unrelated words co‐occurred no more often than expected by chance. Co‐occurrence rates were much higher for words taken from the semantically related sets. In general, concrete words co‐occurred more often than abstract words. This presumably reflects the greater semantic diversity of abstract words (Hoffman, Lambon Ralph, & Rogers, 2013), which means that they occur in wider variety of contexts and consequently appear less frequently with their semantic neighbours.

Within the concrete words, it is interesting to note that items classed as similar co‐occurred more often than those that were associated. This has two implications. First, it suggests that lexical co‐occurrence is an important additional factor for understanding why similarity influences concrete word processing to a greater degree than association. It is likely that similar concrete items benefit from a double whammy of both sharing semantic features and regularly occurring together in language. Recent work also suggests that, in addition to frequent lexical co‐occurrence, objects from the same semantic category regularly co‐occur in real environments (Sadeghi, McClelland, & Hoffman, 2015). Second, it suggests that the distinction between semantic similarity and semantic association is not as clear‐cut as originally thought (at least insofar as association is indexed by lexical co‐occurrence). While early studies treated similarity and association as mutually exclusive, more recent studies have found that items classified as similar tend to be also rated as highly associated (Crutch & Warrington, 2010). Perhaps this is not so surprising. Objects with similar properties are commonly found in the same environments because they share characteristics that make them particularly suited to those environments (e.g., ducks and swans on a lake or bowls and plates in a kitchen).

In contrast, as shown in Figure 4, abstract words were more likely to co‐occur when they were associated. Again, this suggests that rates of lexical co‐occurrence may be an important factor in understanding why associated abstract words prime and interfere with each other more effectively than similar/synonymous abstract words. It also provides empirical support for Crutch and Jackson's (2011) assertion that semantically similar abstract words co‐occur relatively infrequently because their meanings are sufficiently redundant that only one is needed in any given instance. To illustrate this idea, imagine you are talking with a colleague about your latest brilliant idea. You begin a sentence ‘How shall we test this…’. There are a number of different candidate words that could complete this sentence (theory, hypothesis, idea, premise), all with highly similar meanings. It would be unnecessarily verbose to use all of them; instead, the language system is required to select one of these words for production and then avoid producing the others. In other words, production of one word reduces the probability that its synonyms will be produced shortly after and, paradoxically, the more similar the meanings of two words, the less likely it is that both need to be produced. A similar situation arises in comprehension, where the presence of one word in a sentence means that closely synonymous words are unlikely to be encountered immediately afterwards. Importantly, this is not true for words with associated meanings: When *test* is encountered in a sentence, there is a high probability that *theory* will follow shortly after. The analysis of lexical co‐occurrence rates in Figure 4 supports this idea that processing of a particular abstract word primes the language system to expect associated words to occur soon but not words with similar meanings. This could then explain why synonymous abstract words do not interfere with one another in Crutch *et al*.'s studies but associated words do. Critically, this situation does not hold for concrete words because (1) unlike abstract words, semantically similar concrete terms are rarely sufficiently synonymous to be interchangeable and (2) as discussed earlier, similar concrete items *do* frequently co‐occur in language and in the real world.

To summarize, the differential frameworks hypothesis provides a new perspective on the factors that determine how concrete and abstract concepts are structured. Based mainly on studies of priming and interference effects in aphasic patients, Crutch and Warrington (2005, 2007, 2010) have argued that the strength of the relationships between concrete concepts is determined primarily by their conceptual similarity while abstract concepts are related to one another primarily by association. Here, I have presented analyses suggesting that lexical co‐occurrence may play an important role in producing these effects. Concrete terms are more likely to co‐occur in language if they have similar properties, rather than merely being associated, while abstract terms with associated meanings co‐occur more frequently than those with synonymous meanings. One explanation for these findings is that when the meanings of two abstract words are sufficiently similar, they become redundant and only one is used in any given situation (Crutch & Jackson, 2011). The differential frameworks hypothesis makes no commitment as to the neural basis of the differing organization of concrete and abstract words. However, other researchers have proposed, based on work exclusively with concrete concepts, that similarity relations and associative relations are represented in different sites in the brain (Kalénine *et al*., 2009; Mirman & Graziano, 2012; Schwartz *et al*., 2011). Other neuroimaging evidence does not support these claims, however (Jackson, Hoffman, Pobric, & Lambon Ralph, 2015), and a full understanding of how these types of relation are supported neurally remains a major challenge.

### Conclusion

Studies of language‐impaired patients, allied with functional neuroimaging investigations, have made major contributions to our understanding of the cognitive and neural bases of concrete and abstract word comprehension. The fact that disorders can preferentially impair processing of either concrete or abstract words indicates that their representations rely on partially distinct neural systems. Neuroimaging data support the idea that representations of concrete words are rooted more closely in sensory experience and those of abstract words in their use in language. More recently, evidence has emerged for the differential dependence of abstract words on affective, social, and magnitude‐based information. At the same time, the vast majority of patients show greater deficits for abstract words and this attests to the greater processing demands posed by these words. This difficulty most likely arises in part from impoverished representations, relative to concrete concepts, but also from their greater contextual variability, which means that elucidating a contextually appropriate meaning for them requires executive regulation of the semantic system. Finally, the principles governing the organization of concepts may also differ across word types, with concrete words more strongly influenced by semantic similarity while abstract words are more affected by association. The cause of this difference is not yet understood, although it appears to be linked with different patterns of lexical co‐occurrence. Ultimately, it is clear that the disconnection of abstract words from external sensory‐motor experiences has a diverse set of consequences for the cognitive processing of these words. Through exploring these consequences, we gain valuable insights into the structure and operation of the semantic system as a whole.

## Footnotes

1 The original Jefferies *et al*.'s test also featured a middle condition comprising medium imageability words, but as this gave intermediate results, it is not discussed here.

2 The average effect in age‐matched control subjects was 3%.

3 This variation is often termed ‘polysemy’ in the psycholinguistic literature and is a subject of ongoing debate. A full discussion of these issues is beyond the scope of this paper. In brief, however, some researchers hold that there is a distinct semantic representation for each sense of a word (Klein & Murphy, 2001, 2002). Others have argued that the brain does not store discrete senses for words and that variation in the meaning of a word is better characterized a continuous phenomenon (Cruse, 1986; Hoffman, Lambon Ralph, & Rogers, 2013; Hoffman & Woollams, in press; Landauer, 2001). On this view, whenever the context for a word changes, the meaning of the word changes with it, and this graded variation in meaning cannot be captured by assigning each use to one of a small number of different senses.
